# Comparison of functional and morphologic changes between brolucizumab and faricimab in neovascular age-related macular degeneration

**DOI:** 10.1007/s00417-023-06241-8

**Published:** 2023-09-26

**Authors:** Maiko Maruyama-Inoue, Yasuo Yanagi, Tatsuya Inoue, Kazuaki Kadonosono

**Affiliations:** Department of Ophthalmology and Micro-technology, Yokohama City University Medical Center, 4-57 Urafune-cho, Minami-ku, Yokohama, Japan

**Keywords:** Age-related macular degeneration, Polypoidal choroidal vasculopathy, Brolucizumab, Faricimab, Intravitreal injection

## Abstract

**Purpose:**

This study aimed to compare functional and morphologic changes in the loading phase between patients with treatment-naïve macular neovascularization (MNV) due to neovascular age–related macular degeneration (nAMD) treated with either intravitreal brolucizumab (IVBr) or intravitreal faricimab (IVF) injections in a clinical setting.

**Methods:**

We retrospectively studied 92 consecutive eyes of 90 patients with neovascular nAMD who were scheduled to receive IVBr (42 eyes of 41 patients) or IVF (50 eyes of 49 patients) injections between October 2021 and December 2022. All patients received three consecutive monthly injections of 6.0 mg/0.05 mL brolucizumab or 6.0 mg/0.05 mL faricimab. The best-corrected visual acuity (BCVA), central foveal thickness (CFT), and central choroidal thickness (CCT) at baseline and 1, 2, and 4 months after the initial treatment were measured and compared between the groups.

**Results:**

Thirty-seven eyes in IVBr group and forty-seven eyes in IVF group who finished treatments in the loading phase were assessed at the follow-up examination. The BCVA, CFT, and CCT changed significantly after loading phase in both groups (*P* < 0.05 for both comparisons). The IVBr group had more rapid improvement of the BCVA (*P* = 0.037) at 1 month than the IVF group, but there was no difference at 4 months (*P* = 0.367). The CFT and CCT decreases tended to be greater in the IVBr group than in the IVF group throughout the follow-up period. Of the five eyes excluded from the IVBr group, one eye (2.4%) each had intraocular inflammation (IOI) and was a non-responder, and two eyes (4.8%) had retinal pigment epithelial tears after treatment. Of the three eyes excluded from the IVF group, two eyes (4.0%) did not respond to the treatment.

**Conclusions:**

Both IVBr and IVF injections were well-tolerated and improved the VA in treatment-naïve patients with MNV due to nAMD after a loading phase, although IVBr caused a trend toward faster visual improvements in the BCVA. The IVBr group also had greater reductions of the CFT and CCT than the IVF group. However, the potential for adverse events and no response to treatment with each drug are considerations.


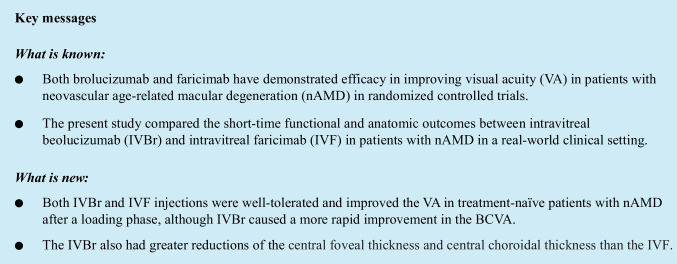


## Introduction

Macular neovascularization (MNV) due to neovascular age–related macular degeneration (nAMD) is most frequently treated with anti-vascular endothelial growth factor (VEGF) agents that have demonstrated efficacy in improving visual acuity outcomes [1, 2]. Polypoidal choroidal vasculopathy (PCV), which is commonly seen in Asian individuals and is regarded as a subtype of nAMD, was defined recently as a variant of type 1 MNV with polyps detected by indocyanine green angiography (ICGA) [3]. The benefits of anti-VEGF agents in patients with PCV have been described [4, 5]. However, frequent injections and financial costs represent significant burdens for patients and medical staff. To reduce these treatment burdens, brolucizumab (Beovu, Novartis International, Basel, Switzerland), an approximate 26-kDa single-chain antibody fragment, is being used to manage nAMD and patients were maintained on every-12-week dosing intervals through 1 year in the phase 3, multicenter, randomized, double-masked trials HAWK and HARRIER studies [6]. Brolucizumab exhibited better fluid control than aflibercept (Eylea, Regeneron Pharmaceuticals, Tarrytown, NY, USA) in the study. However, an independent Safety Review Committee reported that 4.6% of patients in the HAWK and HARRIER trials treated with brolucizumab developed intraocular inflammation (IOI) [7].

Recently, faricimab (Vabysmo, Roche/Genentech, Basel, Switzerland), a bispecific antibody that acts through dual inhibition of angiopoietin-2 (Ang-2) and VEGF-A, was approved to treat nAMD [8]. Treatment intervals up to 16 weeks in the maintenance phase showed similar visual benefits of bimonthly intravitreal aflibercept, indicating the potential to decrease the treatment burden [8]. However, the efficacy of intravitreal faricimab (IVF) in a clinical setting remains uncertain.

While the choice between intravitreal brolucizumab (IVBr) and IVF to treat MNV due to nAMD is an important issue, the two drugs have not been compared. The present study compared the short-time functional and anatomic outcomes between IVBr and IVF in patients with MNV due to nAMD in routine clinical practice.

## Methods

We retrospectively studied 92 eyes of 90 consecutive Japanese patients aged 50 years or older who were newly diagnosed with MNV due to nAMD, including PCV, and who had provided written informed consent for treatment. All patients were initially treated at Yokohama City University Medical Center between October 2021 and December 2022 and followed for 4 months. Brolucizumab was administered from October 2021 to May 2022, and faricimab was administered from June 2022 to December 2022. The study was conducted according to the principles of the Declaration of Helsinki and was approved by the Ethics Committee of Yokohama City University Medical Center.

The inclusion criteria were a diagnosis of MNV due to nAMD, based on clinical, spectral-domain optical coherence tomography (SD-OCT) and angiographic findings and a baseline best-corrected visual acuity (BCVA) of 20/400 or better.

Patients who had previously received treatment for MNV (i.e., laser photocoagulation, submacular surgery, photodynamic therapy, or intravitreal injections of other anti-VEGF agents) or who underwent vitrectomy were excluded. Furthermore, patients with MNV as a result of high myopia, angioid streaks, hereditary disorders, uveitis, or other secondary diseases also were excluded.

All patients received three consecutive monthly injections of 6.0 mg/0.05 mL brolucizumab or 6.0 mg/0.05 mL faricimab as induction therapy. If patients had a complication such as IOI or retinal pigment epithelial (RPE) tears during the induction phase, they were excluded. Patients who had no response to the treatment and were switched to other anti-VEGF agents also were excluded. Non-responder was defined that exudative changes still persisted and central foveal thickness (CFT) did not change (< 5%) or increased over treatment with IVBr or IVF.

The decimal BCVA measured was converted to logarithm of the minimum angle of resolution (logMAR) equivalents for the statistical analysis. The logMAR BCVA, CFT, and central choroidal thickness (CCT) at baseline and 1, 2, and 4 months after initial treatment were measured. If patients treated with faricimab had exudative changes at 2 months, they were evaluated at 3 months and data for the 4-month visit were imputed using the 3 months’ data for the statistical analysis. The CFT was defined as the distance between the internal limiting membrane and Bruch’s membrane at the fovea. The CCT was defined as the thickness between Bruch’s membrane and the inner surface of the choroidal-scleral junction at the fovea.

The primary outcome measure was the comparison of the changes in the BCVA, CFT, and CCT between the two groups. The secondary outcome measures were the changes in the proportions of fluid and dry macula in each group. We evaluated these outcomes in patients with/without PCV.

Digital simultaneous fluorescein angiography and ICGA using a confocal scanning laser ophthalmoscope (SPECTRALIS Product Family Version 5.3; Heidelberg Engineering Inc., Dossenheim, Germany) were performed in a standard manner to diagnose the lesion subtypes. SD-OCT (SPECTRALIS Product Family Version 5.3; Heidelberg Engineering, Germany) was used to evaluate lesion changes during the follow-up period.

For statistical analyses, the baseline characteristics were compared using the unpaired *t*-test and Fisher’s exact tests. The BCVAs, CFTs, and CCTs before and after treatment were compared in each group by the one-way ANOVA with Bonferroni correction. The comparisons of the changes in the three parameters at each timepoint between the two groups were compared using the unpaired t-test. All statistical analyses were performed using Ekuseru-Toukei 2012 (Social Survey Research Information Co., Ltd., Tokyo, Japan). *P* < 0.05 was considered statistically significant.

## Results

### Patient characteristics

Five eyes in the IVBr group and three in the IVF group did not complete the follow-up period and were excluded, leaving 37 eyes (36 patients; 27 men, 9 women; age range, 52–88 years; mean age ± standard deviation [SD], 75.3 ± 8.0 years) in the IVBr group and 47 eyes (46 patients; 27 men, 19 women; age range, 50-89 years; mean age ± SD, 75.4 ± 8.9 years) in the IVF group. The baseline patient characteristics and clinical data are shown in Table 1.

**Table 1:** Patient characteristics of all study eyes

	IVBr group	IVF group	*P*-value
Number of eyes	37	47	
Number of patients	36	46	
Male/Female	27/9	27/19	0.161^a^
Age, mean ± SD, year (range)	75.3 ± 8.0 (52–88)	75.4 ± 8.9 (50–89)	0.897^a^
AMD subtype (non-PCV/PCV) (%)	23/14(62/38)	35/12(74/26)	0.245^b^
Baseline logMAR BCVA	0.42 ± 0.39	0.36 ± 0.33	0.462^a^
Mean CFT ± SD (μm)	517 ± 282	407 ± 187	0.036^a^
Mean CCT ± SD (μm)	193 ± 97	211 ± 95	0.407^a^
Presence of intraretinal fluid, present/none (%)	6/31(16/84)	13/34(28/72)	0.295^b^
Presence of subretinal fluid, present/none (%)	30/7(81/19)	40/7(85/15)	0.770^b^
Presence of PED, present/none (%)	30/7(81/9)	35/12(75/25)	0.601^b^
Presence of SHRM, present/none (%)	18/19(49/51)	24/23(51/49)	1.000^b^

*SD* standard deviation, *AMD* age-related macular degeneration, *PCV* polypoidal choroidal vasculopathy, *logMAR* logarithm of the minimum angle of resolution, *BCVA* best-corrected visual acuity, *CFT* central foveal thickness, *CCT* central choroidal thickness, *PED* pigment epithelial detachment, *SHRM* subretinal hyperreflective materials

^a^*P*-value calculated using the unpaired *t* test

^b^*P*-value calculated using Fisher’s exact test

In the IVBr group, 23 eyes (62.2%) had no PCV and 14 eyes (37.8%) did. Among the 47 eyes in the IVF group, 35 eyes (74.5%) had no PCV and 12 eyes (25.5%) did. Comparison of the groups showed a significant (*P* = 0.036) difference only in the baseline CFT between the two groups.

### VA outcomes

**Fig. 1:**
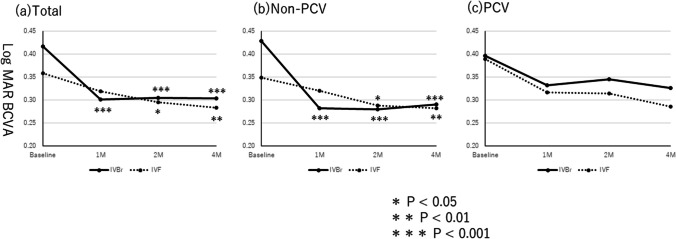
**a** The BCVAs in nAMD with brolucizumab and faricimab. The BCVA outcomes of all cases. Both groups had significant BCVA improvements at 4 months (IVBr group, *P* < 0.001; IVF group, *P* = 0.002). The IVBr group had more rapid BCVA improvement at 1 month than the IVF group (*P* = 0.037) but with no difference at 4 months (*P* = 0.367). **b** The BCVA changes between the IVBr and IVF groups in patients with non-PCV. The IVBr group had a significant BCVA improvement (*P* < 0.001 at 1, 2, and 4 months, respectively). The logMAR BCVA in the IVF group improved significantly compared with baseline post-injection at 2 and 4 months, but not at 1 month (*P* = 0.947, *P* = 0.017, and *P* = 0.006 at 1, 2, and 4 months, respectively). **c** The changes in BCVA between IVBr and IVF in patients with PCV. The BCVA improvement did not reach significance, but showed a trend toward greater visual improvements in both groups

The mean logMAR BCVAs at baseline and 1, 2, and 4 months after the initial injection were 0.42 ± 0.39, 0.30 ± 0.31, 0.31 ± 0.34, and 0.30 ± 0.36 in the IVBr group and 0.36 ± 0.33, 0.32 ± 0.30, 0.30 ± 0.31, and 0.28 ± 0.32 in the IVF group, respectively. In the IVBr group, the post-injection BCVA improved significantly compared with baseline throughout the 4-month period (*P* < 0.001 at 1, 2, and 4 months, respectively). In the IVF group, the post-injection logMAR BCVA at 2 and 4 months, but not at 1 month, improved significantly compared with baseline (*P* = 0.371, *P* = 0.016, and *P* = 0.002 at 1, 2, and 4 months, respectively) (Fig. 1). The IVBr group had more rapid BCVA improvement (*P* = 0.037) at 1 month than the IVF group, with no difference at 4 months (*P* = 0.367).

The clinical courses of each disease subtype are shown in Fig. 1. In patients without PCV, in the IVBr group, the mean logMAR BCVAs at baseline and 1, 2, and 4 months after the initial treatment were 0.43 ± 0.44, 0.28 ± 0.32, 0.28 ± 0.34, and 0.29 ± 0.36 and in the IVF group, 0.35 ± 0.32, 0.32 ± 0.31, 0.29 ± 0.32, and 0.28 ± 0.31, respectively. In the IVBr group, the post-injection BCVA improved significantly compared with baseline throughout the follow-up period (*P* < 0.001 at 1, 2, and 4 months, respectively). In the IVF group, the post-injection logMAR BCVA at 2 and 4 months, but not at 1 month, improved significantly compared with baseline (*P* = 0.947, *P* = 0.017, and *P* = 0.006 at 1, 2, and 4 months, respectively). The IVBr group had more rapid BCVA improvement (*P* = 0.010) at 1 month than the IVF group, with no difference at 4 months (*P* = 0.108).

In patients with PCV, the mean logMAR BCVAs at baseline and 1, 2, and 4 months after initial treatment were 0.40 ± 0.31, 0.33 ± 0.31, 0.35 ± 0.35, and 0.33 ± 0.38 in the IVBr group and 0.39 ± 0.35, 0.32 ± 0.27, 0.32 ± 0.31, and 0.29 ± 0.35 in the IVF group, respectively. In both groups, the post-injection BCVA did not improve significantly compared with the preoperative VA throughout the 4-month period (*P* = 0.634, *P* = 1.000, *P* = 0.465 at 1, 2, and 4 months in the IVBr group and *P* = 1.000, *P* = 1.000, *P* = 0.582 at 1, 2, and 4 months in the IVF group) (Fig. 1). There were no significant differences in the BCVA improvement between the two groups at any time (*P* > 0.05 for all)

### Comparison of CFT

**Fig. 2:**
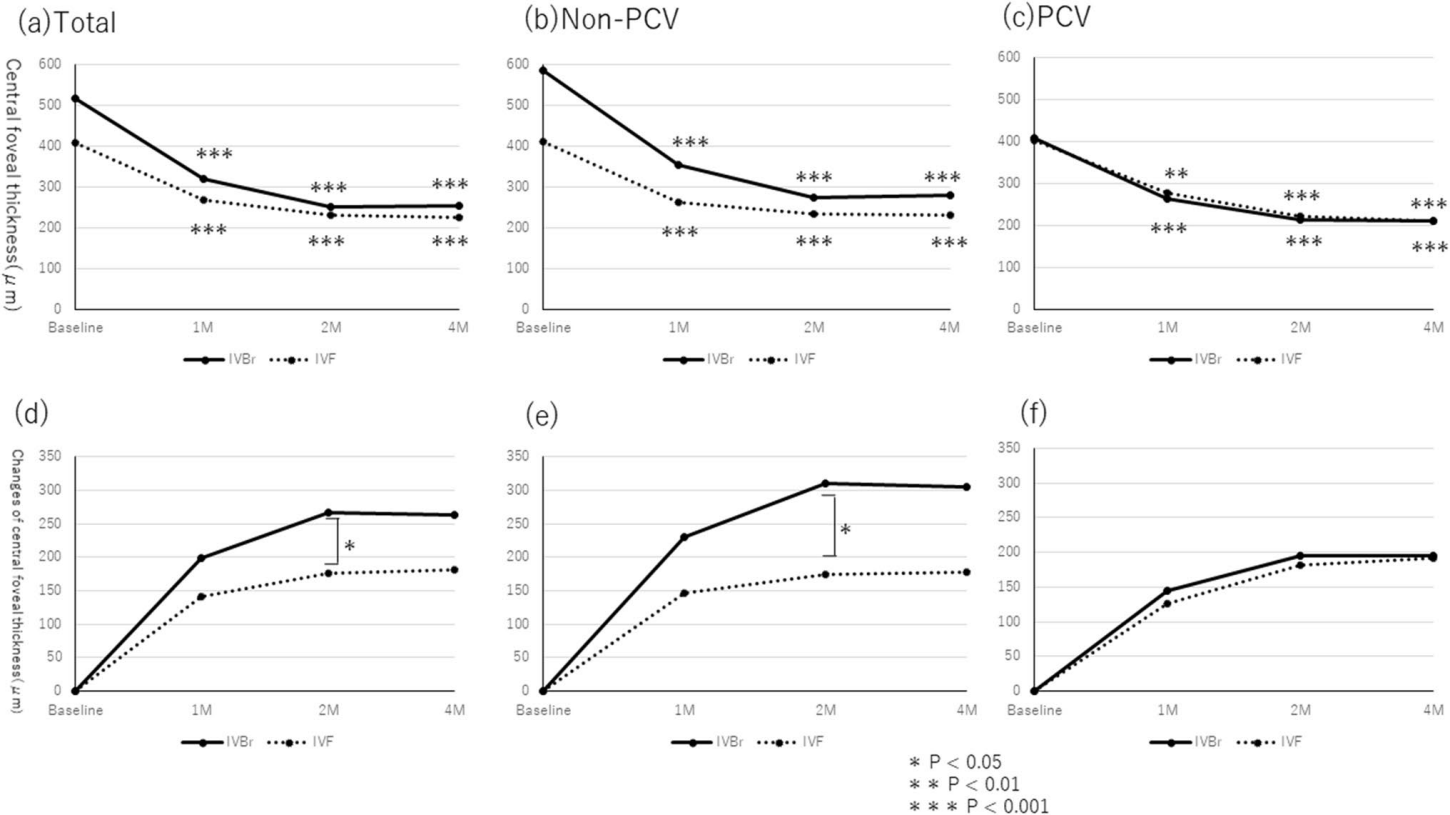
**a** Changes in the CFTs in patients with nAMD treated with brolucizumab and faricimab**.** The CFT outcomes of all cases. The mean CFTs at 1, 2, and 4 months decreased significantly from baseline in both groups (*P* < 0.001 for all). **b** The CFT outcomes in patients without PCV showed that the mean CFTs at 1, 2, and 4 months decreased significantly from baseline in both groups (*P* < 0.001 for all). **c** The CFT outcomes in patients with PCV showed that the mean CFTs at 1, 2, and 4 months decreased significantly from baseline in both groups (*P* < 0.001 for all in the IVBr group and *P* = 0.002, *P <* 0.001, and *P* < 0.001 at 1, 2, and 4 months in the IVF group). **d** The changes in CFT between IVBr and IVF in all cases. Significant differences in the degrees of decrease in CFTs are observed between the two groups only at 2 months (*P* = 0.118, *P* = 0.044, and *P* = 0.089, at 1, 2, and 4 months). **e** The changes in the CFTs between IVBr and IVF in patients without PCV. Significant differences in the degree of the CFT decrease are seen between the two groups only at 2 months (*P* = 0.092, *P* = 0.027, and *P* = 0.057, at 1, 2, and 4 months). **f** The CFT changes between IVBr and IVF in patients with PCV. No significant difference in CFT improvement is seen between the two groups at each time point (*P* = 0.650, *P* = 0.797, and *P* = 0.933 at 1, 2, and 4 months)

In the IVBr group, the mean CFTs at baseline and 1, 2, and 4 months after the initial injection were 517 ± 282, 318 ± 190, 250 ± 130, and 253 ± 124 μm, respectively; in the IVF group, the respective values were 407 ± 187, 266 ± 106, 231 ± 98, and 226 ± 94 μm. In both groups, the mean CFTs at 1, 2, and 4 months decreased significantly compared with baseline (*P* < 0.001 for all) (Fig. 2). In each disease subtype, the post-injection CFT also decreased significantly in both groups during follow-up (*P* < 0.01 for all) (Fig. 2).

In total, the mean differences in the pre-injection and post-injection CFTs at 1, 2, and 4 months after initial treatment were 198 ± 198, 267 ± 250, and 264 ± 272 μm in the IVBr group and 141 ± 133, 177 ± 151, and 182 ± 160 μm in the IVF group, respectively. The CFTs in the IVBr group improved more than the IVF group at 2 months, not at 1 and 4 months after the initial treatment (*P* = 0.118, *P* = 0.044, and *P* = 0.089, respectively) (Fig. 2).

In patients without PCV, the mean differences in the pre-injection and post-injection CFTs at 1, 2, and 4 months after initial treatment were 231 ± 238, 311 ± 297, and 305 ± 325 μm in the IVBr group and 145 ± 138, 172 ± 155, and 173 ± 167 μm in the IVF group, respectively. More improvement in the CFT was seen at 2 months, not at 1 and 4 months, after the initial treatment between the groups (*P* = 0.092, *P* = 0.027, and *P* = 0.057, respectively) (Fig. 2).

In patients with PCV, the mean differences in pre-injection and post-injection CFTs at 1, 2, and 4 months after the initial treatment were 145 ± 86, 195 ± 121, and 196 ± 134 μm in the IVBr group and 126 ± 126, 182 ± 146, and 191 ± 144 μm in the IVF group, respectively. No significant difference in CFT improvement was observed between the two groups at each timepoint (*P* = 0.650, *P* = 0.797, and *P* = 0.933 at 1, 2, and 4 months) (Fig. 2).

### Comparison of CCT

**Fig. 3:**
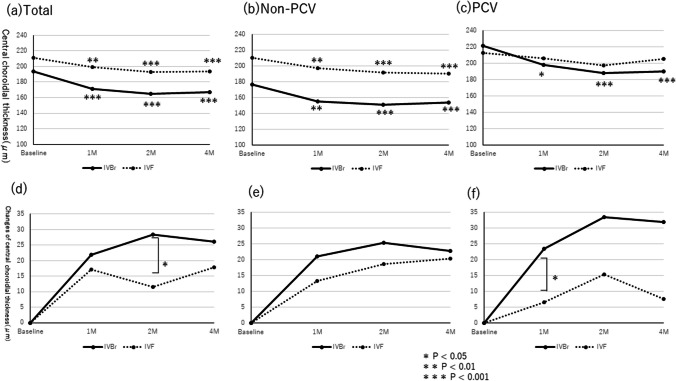
**a** The CCT changes in patients with nAMD treated with brolucizumab and faricimab. The CCT outcomes of all cases showed that the mean CCT at 1, 2, and 4 months decreased significantly from baseline in both groups (*P* < 0.001 for all in the IVBr group and *P* = 0.002, *P* < 0.001, and *P* < 0.001 at 1, 2, and 4 months in the IVF group). **b** The CCT outcomes in patients without PCV. The post-injection CCTs also decreased significantly in both groups during the follow-up period (*P* = 0.002, *P* < 0.001, and *P* < 0.001 at 1, 2, and 4 months in the IVBr group and *P* = 0.002, *P* < 0.001, and *P* < 0.001 at 1, 2, and 4 months in the IVF group). **c** The CCT outcomes in patients with PCV showed that the post-injection CCT decreased significantly in the IVBr group during the follow-up period (*P* = 0.024, *P* < 0.001, and *P* < 0.001 at 1, 2, and 4 months, respectively). In the IVF group, no significant decrease in CCT was observed at each timepoint (*P* = 1.000, *P* = 0.228, and *P* = 1.000 at 1, 2, and 4 months). **d** The changes in CCT between IVBr and IVF in all cases. Significant differences in the degrees of the CCT decreases are seen between the two groups only at 2 months (*P* = 0.184, *P* = 0.030, and *P* = 0.075, at 1, 2, and 4 months, respectively). **e** The changes in CCTs between IVBr and IVF in patients without PCV. No greater degree of CCT improvement after the initial treatment at 1, 2, and 4 months is seen between the groups (*P* = 0.211, *P* = 0.382, and *P* = 0.748, respectively). **f** The changes in CCTs between IVBr and IVF in patients with PCV. Greater CCT improvement is seen after the initial treatment only at 1 month between the two groups (*P* = 0.020, *P* = 0.058, and *P* = 0.107, respectively)

In the IVBr group, the mean CCTs at baseline and 1, 2, and 4 months after the initial injection were 193 ± 97, 171 ± 87, 165 ± 83, and 167 ± 83 μm, respectively. In the IVF group, the respective values were 211 ± 95, 199 ± 94, 193 ± 89, and 194 ± 95 μm. In both groups, the mean CCTs at all times decreased significantly compared with baseline (*P* < 0.001 for all in the IVBr group and *P* = 0.002, *P* < 0.001, and *P* < 0.001 at 1, 2, and 4 months in the IVF group) (Fig. 3).

In patients without PCV, the post-injection CCT decreased significantly in both groups during the follow-up period (*P* < 0.01 for all). In patients with PCV, the post-injection CCT decreased significantly in the IVBr groups during the follow-up period (*P* < 0.05 for all). In the IVF group, no significant decrease in CCT was observed at each timepoint (*P* > 0.05 for all) (Fig. 3).

In total, the mean differences in pre-injection and post-injection CCTs at 1, 2, and 4 months after initial treatment were 22 ± 24, 28 ± 33, and 26 ± 35 μm in the IVBr group and 17 ± 28, 11 ± 19, and 18 ± 20 in the IVF group, respectively. The CCTs improved more in the IVBr group at 2 months, but not at 1 and 4 months after the initial treatment than the IVF groups (*P* = 0.184, *P* = 0.030, and *P* = 0.075 at 1, 2, and 4 months, respectively) (Fig. 3).

In patients without PCV, the mean differences in pre-injection and post-injection CCTs at 1, 2, and 4 months after the initial treatment were 21 ± 26, 25 ± 36, and 23 ± 31 μm in the IVBr group and 13 ± 21, 19 ± 22, and 20 ± 26 μm in the IVF group, respectively. The CCT improvements between the groups were similar at 1, 2, and 4 months after the initial treatment (*P* = 0.211, *P* = 0.382, and *P* = 0.748, respectively) (Fig. 3).

In patients with PCV, the mean differences in pre-injection and post-injection CCTs at 1, 2, and 4 months after initial treatment were 23 ± 22, 33 ± 28, and 32 ± 40 μm in the IVBr group and 7 ± 10, 15 ± 14, and 8 ± 32 μm in the IVF group, respectively. The CCTs improved more in the IVBr group at 1 month, but not at 2 and 4 months after the initial treatment than the IVF group (*P* = 0.020, *P* = 0.058, and *P* = 0.107, respectively) (Fig. 3).

### Comparison of proportion of fluid during the loading phase

**Fig. 4:**
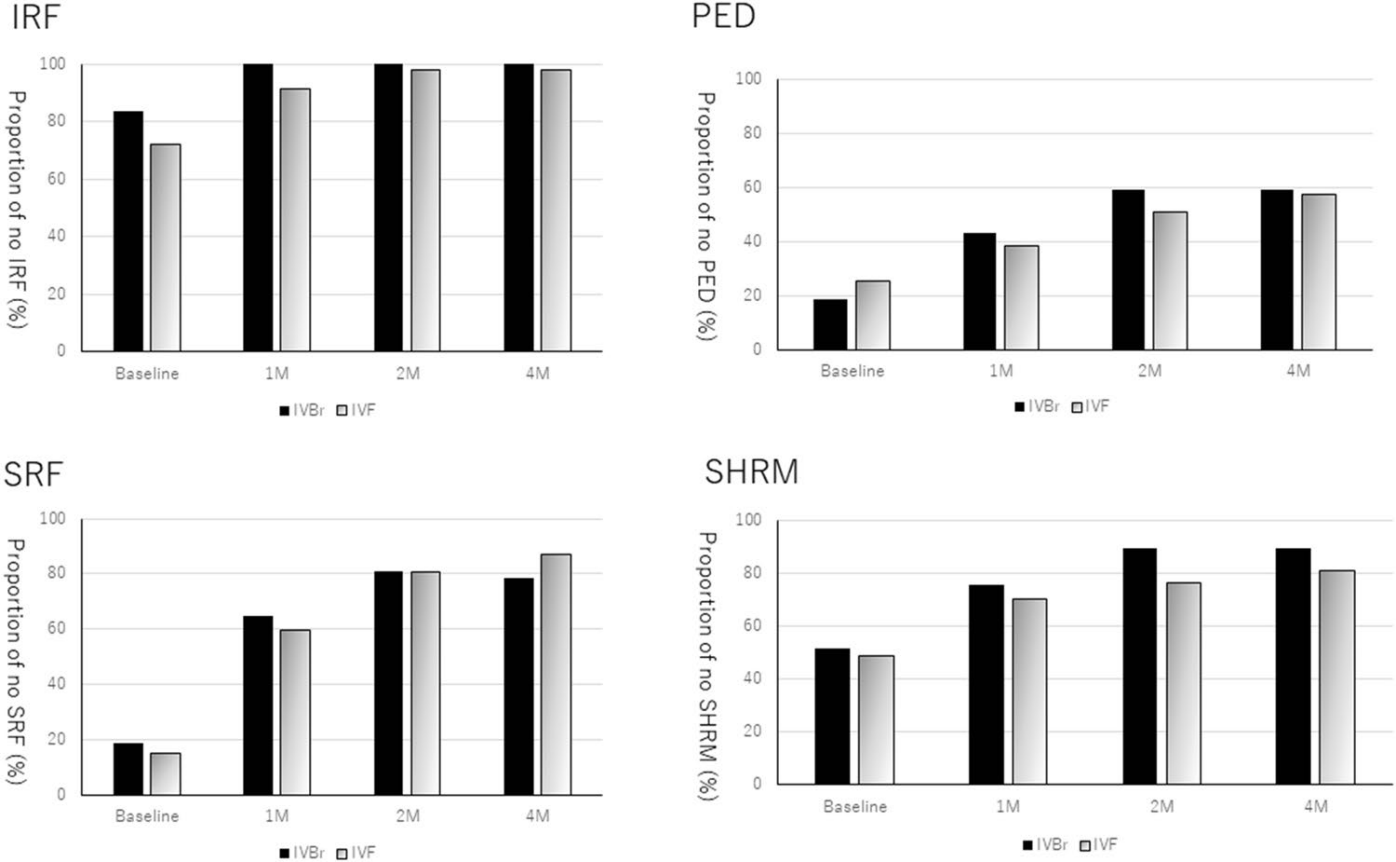
Changes in the proportions of the absence of exudative findings in the IVBr and IVF groups. No significant differences in the proportions of patients with any exudative findings (IRF, SRF, PED, and SHRM) were observed between the IVBr and IVF groups at any time (*P* > 0.05 for all)

The numbers of eyes with no intraretinal fluid (IRF) during the loading phase were 31 (83.8%), 37 (100%), 37 (100%), and 37 (100%) in the IVBr group and 34 (72.3%), 43 (91.5%), 46 (97.9%), and 46 (97.9%) in the IVF group at baseline, 1, 2, and 4 months after the initial treatment. However, the numbers of eyes with no subretinal fluid (SRF) during the loading phase were 7 (18.9%), 24 (64.9%), 30 (81.1%), and 29 (78.4%) in the IVBr group and 7 (14.9%), 28 (59.6%), 38 (80.9%), and 41 (87.2%) in the IVF group at the respective timepoints. The numbers of eyes with no pigment epithelial detachment (PED) during the loading phase were 7 (18.9%), 16 (43.2%), 21 (59.5%), and 21 (59.5%) in the IVBr group and 12 (25.5%), 18 (38.3%), 24 (51.1%), and 27 (57.4%) in the IVF group at baseline, 1, 2, and 4 months after the initial treatment. The numbers of eyes with no subretinal hyperreflective materials (SHRM) during the loading phase were 19 (51.4%), 28 (75.7%), 33 (89.2%), and 33 (89.2%) in the IVBr group and 23 (48.9%), 33 (70.2%), 36 (76.6%), and 38 (80.9%) in the IVF group at baseline, 1, 2, and 4 months after the initial treatment. No significant differences in the proportions of patients with any exudative findings were observed between the groups at any timepoint (*P* > 0.05 for all) (Fig. 4).

**Fig. 5:**
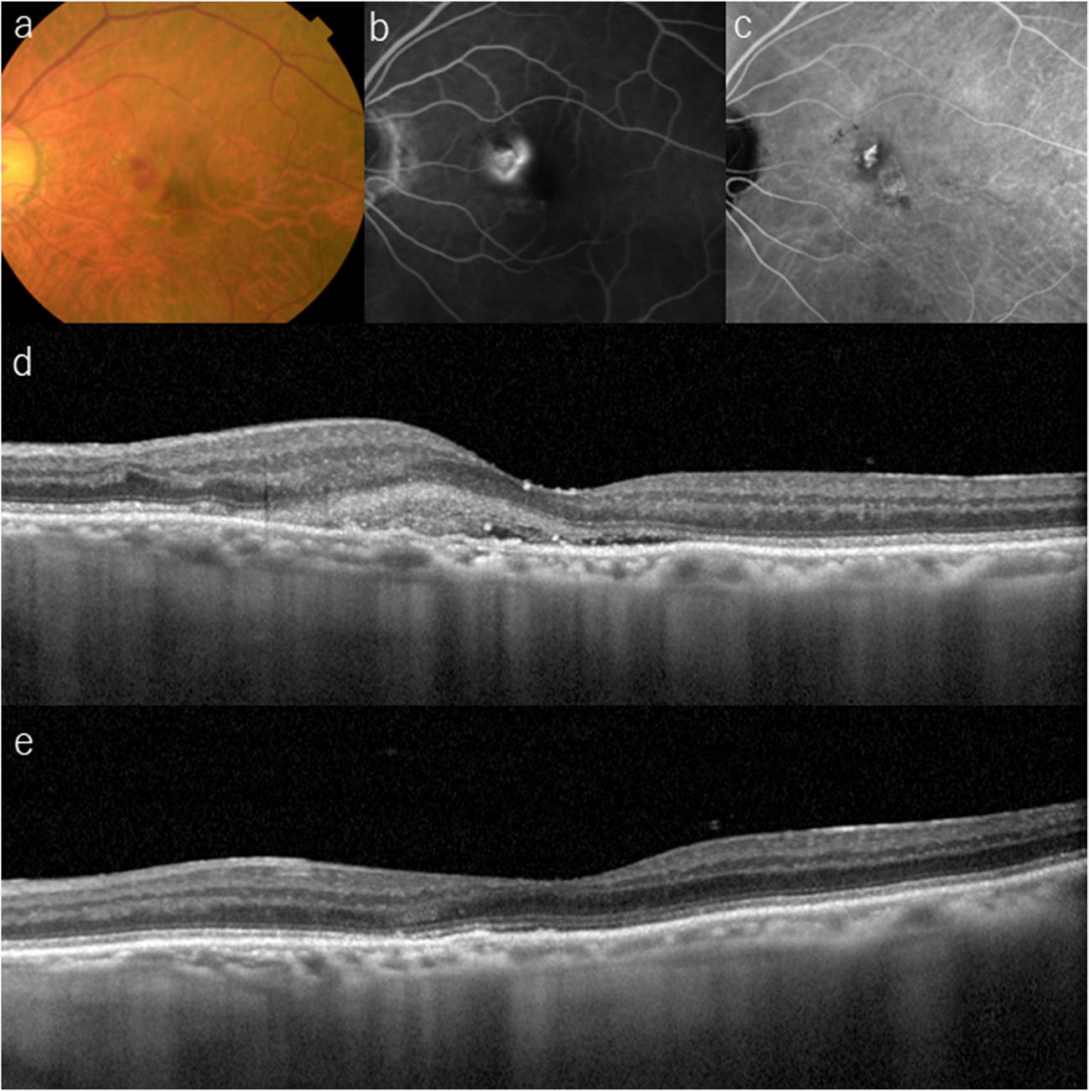
**a** A 75-year-old man presented with visual loss in his left eye (BCVA 20/40). Funduscopic examination showed a white lesion with a macular hemorrhages. **b** Fluorescein angiography shows leakage from the lesion and blockage due to the hemorrhage. **c** ICGA shows a polypoidal lesion and abnormal vascular network. **d** A baseline OCT image shows SHRM due to hemorrhage with subretinal fluid. He was diagnosed with PCV and treated with monthly IVBr injections during the loading phase. **e** Fundus photography at 4 months shows that the exudative changes resolved. His BCVA improved to 20/25

**Fig. 6:**
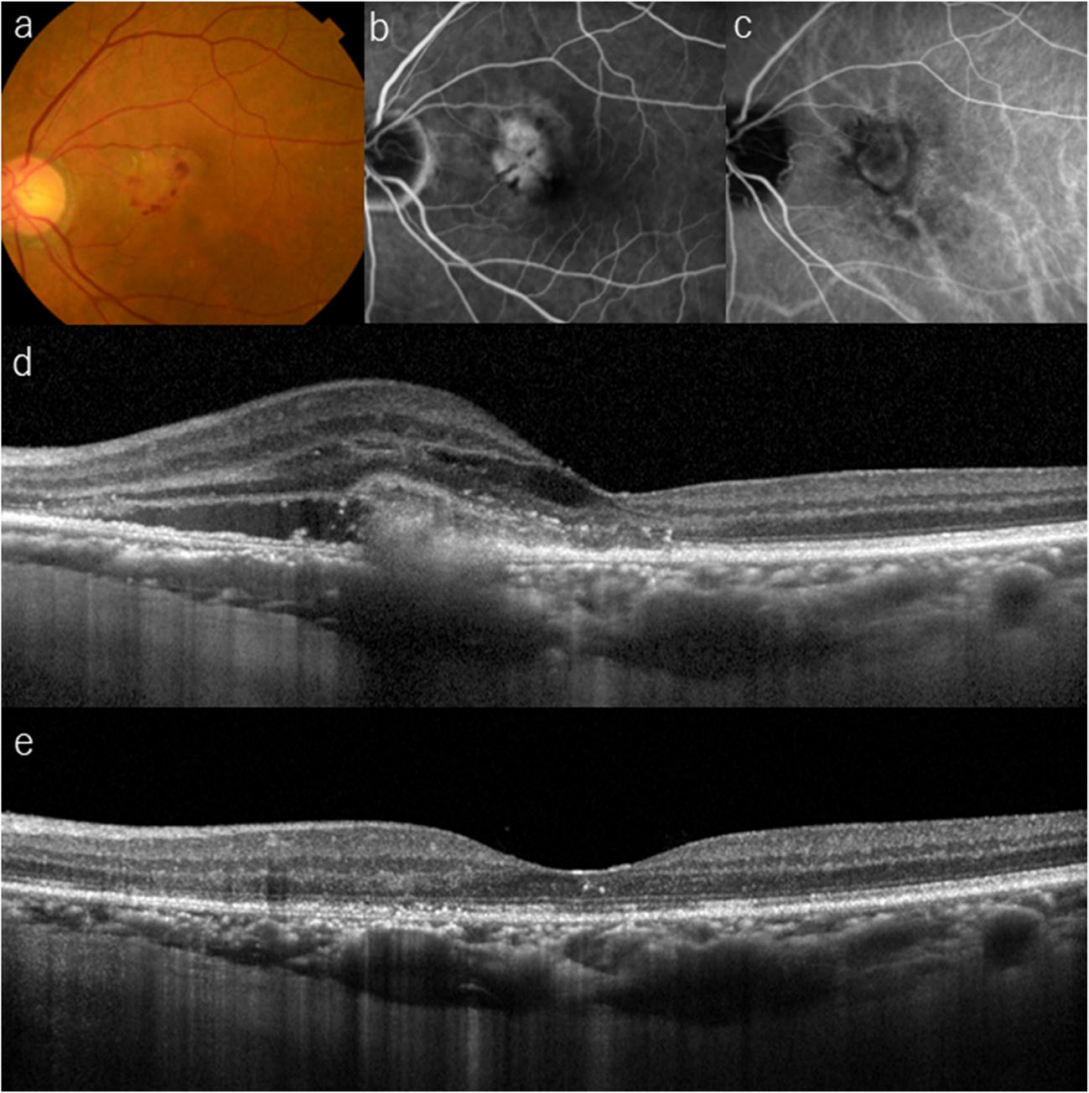
**a** A 72-year-old woman presented with metamorphopsia in her left eye (BCVA 20/50). Funduscopic examination shows a white lesion with a macular hemorrhage. **b** Fluorescein angiography shows leakage from the lesion and blockage due to the hemorrhage. **c** ICGA shows a neovascular lesion. **d** A baseline OCT image shows MNV above the RPE, intraretinal/subretinal fluid, and SHRM. She was diagnosed with type 2 MNV and received three monthly IVF treatment during the loading phase. **e** Fundus photography at 4 months, i.e., 8 weeks after third injection, shows resolved exudative changes. Her BCVA improved to 20/20

A dry macula was achieved in 24 eyes (64.9%) in the IVBr group and 34 eyes (72.3%) in the IVF group 4 months after the initial treatment, with no significant difference (*P* = 0.468). Figures 5 and 6 show the results for eyes in the IVBr and IVF groups, respectively.

### Reason to be excluded

**Fig. 7:**
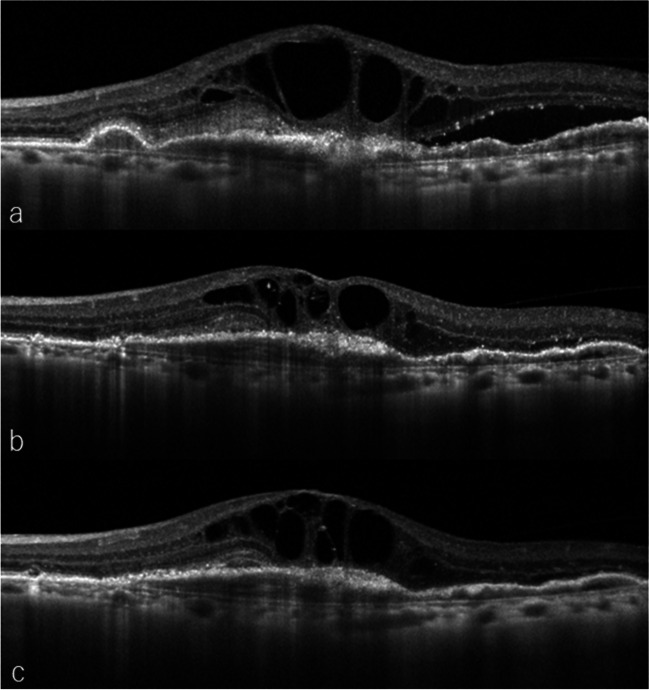
OCT findings of the patient identified as non-responder to faricimab therapy. **a** CFT at baseline was 862 μm. Initial injection of faricimab was performed. **b** At 1 month after the initial treatment, CFT decreased to 521 μm. Second faricimab injection was performed. **c** At 2 months after the initial treatment, fluid persisted and CFT increased to 681 μm. Identified as non-responder to faricimab therapy, the patient was switched to other anti-VEGF agents

Five eyes in the IVBr group and three in the IVF group were excluded. In the IVBr group, one patient (2.4%) stopped attending the outpatient clinic, one eye (2.4%) each had intraocular inflammation (IOI) and was a non-responder, and two eyes (4.8%) had RPE tears after treatment. In the IVF group, one patient (2.0%) stopped attending the outpatient clinic and two eyes (4.0%) were non-responders. No cases had severe systemic complications. Figure 7 shows the findings of the patient identified as non-responder to faricimab therapy.

## Discussion

The current results showed that IVBr and IVF were well-tolerated and improved VA in treatment-naïve patients with nAMD during the loading phase. No significant differences were seen in the proportion of exudative findings at any time. Although the IVBr group had significantly greater visual improvement than the IVF group at 1 month after the initial treatment, no significant differences were seen in the degree of BCVA improvement at 4 months. Furthermore, CFT and CCT decreases tended to be greater in the IVBr group than the IVF group throughout the follow-up period. To our knowledge, the present report is the first to evaluate faricimab in a real-world clinical setting by comparing it with brolucizumab in patients with nAMD. Furthermore, we classified two disease types in the present study, non-PCV and PCV, and evaluated treatment outcomes in both.

In this study, the post-injection BCVAs in both groups improved significantly compared with baseline after three monthly injections. Furthermore, the IVBr group had more rapid BCVA improvement at 1 month than the IVF group. Because the IVBr group showed a slightly worse baseline VA, this may have influenced the outcomes. However, the more rapid BCVA improvement in the IVBr group at 1 month might have resulted from the difference in molecular weight and an affinity for VEGF between the two agents. Faricimab has a 146-kDa novel humanized bispecific immunoglobulin G monoclonal antibody designed for intraocular use with affinity for VEGF-A and blocking Ang-2 [9]. However, as previous report described [10], the lower molecular weight of brolucizumab (26 kDa) might facilitate delivery of more drug per injection compared with other available anti-VEGFs and potentially may have more effective tissue penetration and increased effectiveness. Also, a single-chain variable fragment of brolucizumab is a binding agent independent of a heavy molecular support structure, maintaining its total binding capacity to the target. As a result, greater number of molecules per injection can be administered in the same volume, and more bioavailability in target tissues is attained [11]. This could be responsible for the greater CFT reduction in the IVBr group than in the IVF group throughout the follow-up period. Finally, both treatments achieved visual improvements in patients with nAMD at 2 and 4 months that did not differ significantly. Neutralization of Ang-2 by faricimab may restore vessel-stabilizing effects and reduce inflammatory reactions, resulting in a disease-modifying effect compared with anti-VEGF monotherapy [9, 12]. Therefore, faricimab might take time to be effective longer and gradually show visual improvement in patients with nAMD.

Despite a trend toward visual improvement after treatment in both groups compared with baseline in patients with PCV, these differences did not reach significance possibly because the numbers were not powered to show differences between pre- and post-injection. Spaide et al. described that ICGA imaging showed a branching vascular network and various number of aneurysmal dilations at the outer edge of the expanding lesion [3]. Dansingani et al. also described PCV as “aneurysmal type 1 neovascularization” [13]. Aneurysms occur when a focal weakness in a vessel wall due to atherosclerosis, vessel wall atrophy, inflammation, genetic factors, trauma, and pericyte loss causes elastic decompensation [13]. The potential advantages of faricimab, as an Ang-2 inhibitor, might restore the weakness of the vessel wall of polyps, which result in better functional and anatomical outcomes in the treatment of PCV compared to existing medications.

In our study, the mean differences in the pre-injection and post-injection CFTs at 4 months were 264 ± 272 μm in the IVBr group and 182 ± 160 μm in the IVF group, respectively, which was not significantly different. Although IVBr group tended to have greater reduction of CFT than IVF group, there were no significant differences in the proportion of dry macula at 4 months. On the other hand, in the TENAYA and LUCERNE studies, the mean reduction of CFT at 12 weeks was about 145 μm, which demonstrated a greater reduction in retinal thickness during the loading injection compared to aflibercept (133 μm) [14]. Although a direct comparison between our study and previous results is difficult, CFT reduction using both brolucizumab and faricimab seem to be favorable, compared with aflibercept, in Japanese patients with nAMD during the loading phase.

The CCT significantly decreased in both groups in total, suggesting that both drugs penetrate the choroid and make it thinner, which might affect the choroidal circulation and possibly promote outer retinal atrophy and future declining VA. The CCT reduction tended to be greater in the IVBr group than in the IVF group. Therefore, the effect of IVF on the choroid seems to be smaller than that of IVBr. Intravitreal injection of faricimab may be a better treatment option than IVBr in eyes with a thin choroid, such as retinal angiomatous proliferation, to reduce the risk of atrophy. However, IVBr may be a better choice than IVF in eyes with a thicker choroid such as PCV.

In this study, among the five eyes excluded from the IVBr group, complications such as IOI (2.4%), RPE tears (4.8%), and refractoriness treatment (2.4%) occurred. In the three eyes excluded from the IVF group, two eyes (4.0%) were non-responders. Inflammation can develop after brolucizumab treatment [15]. The Safety Review Committee of the HAWK and HARRIER study showed that the IOI incidence was 4.6% after brolucizumab treatment [7]. However, nearly all patients who developed IOI without retinal vasculitis returned to baseline if brolucizumab injections were stopped and patients were treated promptly [16]. Physicians should be aware of IOI-related events during IVBr treatment; however, carefully consideration should be given to whether patients should receive brolucizumab by balancing treatment advantages and drawbacks. For example, previous reports described that female sex [17, 18], a prior IOI and/or retinal vascular occlusion [18], and old age [19] were the risk factors for emerging IOI after IVBr. Based on these results, faricimab could be prioritized for use in patients who have risk factors for IOI. On the other hand, 4.8% of eyes treated with brolucizumab had RPE tears after treatment, although no patients had them after IVF. Contraction of the choroidal neovascular membrane by anti-VEGF treatments adds tractional forces to the RPE monolayer, which may cause RPE tears. Larger PEDs especially have an increased risk of RPE tears after anti-VEGF therapy with increasing contraction of the choroidal neovascular membrane [20]. In this study, the CFT in the IVBr group was significantly larger than in the IVF group because some patients had large PEDs. We speculated that larger PEDs in the IVBr group caused RPE tears in two patients. Finally, some patients in both groups were non-responders and were switched to other anti-VEGF agents. While most patients had good responses to both treatments, some may not. Although these complications are rare, the potential for adverse events or no response resulting from each treatment should be considered when treatment starts.

The main limitations were the study’s retrospective nature and short-term outcomes. Further prospective studies should involve long-term outcomes. Furthermore, the data collection for faricimab-treated patients was later than that from brolucizumab-treated patients. As a result, significant differences in CFT between the IVBr and IVF groups may have introduced bias into the results. To minimize this, we compared the CFT improvements between the groups and showed a greater CFT reduction in the IVBr group in total.

## Conclusions

IVBr and IVF treatments were well-tolerated and improved the VA of treatment-naïve patients with nAMD during the loading phase. Despite a trend toward faster visual improvements and greater CFT and CCT reductions with IVBr, both treatments showed functional and morphologic improvements after the loading phase.
